# Removing spatial responses reveals spatial concepts—even in a culture with mixed reading habits

**DOI:** 10.3389/fnhum.2014.00966

**Published:** 2014-11-26

**Authors:** Samuel Shaki, Martin H. Fischer

**Affiliations:** ^1^Psychology Department, Ariel UniversityAriel, Israel; ^2^Psychology Department, Potsdam UniversityPotsdam, Germany

**Keywords:** cross-cultural, random number generation, mental number line, embodied numerical cognition, automatic processing

There is currently a renewed discussion about the effects of cultural immersion on our cognitive representation of the world. This discussion is inspired by the notion that sensory and motor experiences are part of all conceptual knowledge (e.g., Barsalou, 2008). The domain of number knowledge is particularly intriguing in this regard because numbers are universal concepts but their use varies strongly across cultures; they occur in different surface forms (logographs, digits, finger postures, number words in various orthographies and with different bases) and are processed in different directions (e.g., East Arabic: right to left; English: left to right; Chinese: top to bottom; Ifrah, 1981).

Rashidi-Ranjbar et al. (2014) recently studied number representations in Farsi readers who read words from right to left but numbers from left to right. Participants generated 80 random numbers while their heads turned alternatingly left or right; contrary to the predicted bias in favor of small numbers when turning right and large numbers when turning left (Loetscher et al., 2008), the authors found no difference between these conditions. Participants did, however, exhibit an attentional bias consistent with their habitual reading direction. The authors interpret their findings in the light of other work (including our own: e.g., Shaki et al., 2009; Shaki and Fischer, 2012) as showing that mixed directional reading habits for text and numbers cancel each other and thus there is “No horizontal numerical mapping in a culture with mixed-reading habits.”

We wish to revise this “cancelation account” and propose to consider their new result and our previous findings as reflecting contributions from both embodied and situated sources (cf. Fischer and Brugger, 2011). Embodiment refers to previous sensory-motor experiences while situatedness encompasses current task demands. In this view, directional reading habits are embodied and lead to the positioning of numbers on a “mental number line” (MNL), thus imbuing it with its spatial orientation. Head turning imposes a situational constraint that directs attention and thus makes particular segments of the MNL more available than others.

The question is: Why did the head turn manipulation fail in Rashidi-Ranjbar et al. (2014)? We propose to account for this by assigning more weights to situated compared to embodied factors when it comes to assessing spatial-numerical associations. We note that all previously employed assessments manipulated space along the horizontal dimension, e.g., by positioning response buttons on the left and right sides, or by requiring participants to adjust their head postures (for review see Fischer and Shaki, 2014). These situated task constraints may have prevented the embodied number representation to emerge because situated factors overrule embodied contributions to current performance. Therefore, it is essential to remove spatial situated task constraints if we wish to properly assess whether there are horizontal spatial-numerical mappings in readers from cultures with mixed reading habits.

Evidence for this proposal comes from two methodological approaches. In one, the conflict between directional scanning habits is avoided by placing the response buttons orthogonal to the conflict-carrying dimension (Shaki and Fischer, 2012). In the other approach, the spatial mapping can even be detected within the conflict-carrying (horizontal) dimension if all spatial features from both the critical stimulus and the response are removed (Fischer and Shaki, under review). Using this latter approach, we asked Hebrew readers to decide whether to press a single button or not, based on a conjunction rule that stated response-relevant features of the stimulus. Stimuli were either digits 1, 2, 8, or 9, or left- or right-facing objects, and response rules were of the form “Press if the object faces left/right or the number is larger/smaller than 5.” Comparing performance across these four conditions, we show that small numbers are indeed associated with left space and larger numbers with right space, without manipulating space along the horizontal dimension. This pattern reveals a left-to-right oriented MNL in Hebrew readers.

To summarize, we argue that the reason for Rashidi et al.'s null result is not the absence of a horizontal MNL but the activation of conflicting spatial codes through head turns along the horizontal dimension. In other words, the left-to-right MNL as a result of directional number processing was embodied even if the situated task constraints concealed its expression. Removing spatial situated task constraints reveals horizontal spatial-numerical mappings, even in readers from cultures with mixed reading habits.

## Conflict of interest statement

The authors declare that the research was conducted in the absence of any commercial or financial relationships that could be construed as a potential conflict of interest.
